# Anemia, diet and therapeutic iron among children living with HIV: a prospective cohort study

**DOI:** 10.1186/s12887-015-0484-7

**Published:** 2015-10-19

**Authors:** Anita Shet, PK Bhavani, N. Kumarasamy, Karthika Arumugam, S. Poongulali, Suresh Elumalai, Soumya Swaminathan

**Affiliations:** Department of Pediatrics, St. John’s Medical College Hospital, Sarjapur Road, Bangalore, 560034 India; Department of Public Health Sciences, Karolinska Institutet, Stockholm, Sweden; Department of Clinical Research, National Institute for Research in Tuberculosis, 1 Sathiyamoorthy Road, Chetput, Chennai India; YRG Center for AIDS Research and Education, Voluntary Health Services Taramani, Chennai, India; Antiretroviral Treatment Center, Institute of Child Health, Egmore, Chennai India; Indian Council of Medical Research (ICMR), New Delhi, India

**Keywords:** HIV, Anemia, Children, Iron deficiency, Anemia of chronic disease, Dietary iron, Iron therapy, India

## Abstract

**Background:**

Children living with HIV have higher-than-normal prevalence of anemia. The beneficial effect of therapeutic iron has been questioned in the setting of high prevalence of infections. This study examines anemia prevalence and effect of standard therapeutic iron on HIV disease progression among children.

**Methods:**

Perinatally-infected children aged 2–12 years were enrolled at three sites in southern India, and were followed for 1 year with clinical assessments, dietary recall and anthropometry. Laboratory parameters included iron markers (ferritin, soluble transferrin receptor) and other micronutrient levels (vitamin A, B_12_, folate). Iron was given to anemic children based on WHO guidelines. Statistical analyses including frequency distributions, chi square tests and multivariate logistic regression were performed using Stata v13.0.

**Results:**

Among 240 children enrolled (mean age 7.7 years, 54.6 % males), median CD4 was 25 %, 19.2 % had advanced disease, 45.5 % had malnutrition, and 43.3 % were on antiretroviral treatment (ART) at baseline. Anemia was prevalent in 47.1 % (113/240) children. Iron deficiency was present in 65.5 %; vitamin A and vitamin B_12_ deficiency in 26.6 % and 8.0 % respectively; and anemia of inflammation in 58.4 %. Independent risk factors for anemia were stunting, CD4 < 25 %, detectable viral load ≥400 copies/ml and vitamin A deficiency. Inadequate dietary iron was prominent; 77.9 % obtained less than two-thirds of recommended daily iron. Among clinically anemic children who took iron, overall adherence to iron therapy was good, and only minor self-limiting adverse events were reported. Median hemoglobin rose from 10.4 g/dl to 10.9 mg/dl among those who took iron for 3 months, and peaked at 11.3 mg/dl with iron taken for up to 6 months. Iron was also associated with a greater fall in clinical severity of HIV stage; however when adjusted for use of ART, was not associated with improvement in growth, inflammatory and CD4 parameters.

**Conclusions:**

Children living with HIV in India have a high prevalence of anemia mediated by iron deficiency, vitamin A deficiency and chronic inflammation. The use of therapeutic iron for durations up to 6 months appears to be safe in this setting, and is associated with beneficial effects on anemia, iron deficiency and HIV disease progression.

## Background

Anemia is a common co-morbid condition among HIV-infected children and has a profound impact on disease progression and mortality [1–4]. A review of this topic indicated that anemia prevalence is higher among HIV-infected children compared to HIV-uninfected children in both high and low-income settings [5]. Since anemia and malnutrition are reported in over 50 % of HIV-infected children in low-income settings [6–9], it is important to understand further the etiology and risk factors for development of anemia. The practice of giving iron to HIV-infected children is based on weak evidence, particularly in areas with high prevalence of HIV, anemia and malaria [10]. Routine use of iron in malaria-endemic settings has been shown to have detrimental effects, particularly among those children who are not iron deficient [11]. Understanding the role of iron therapy in anemia has important implications for the clinical evaluation and treatment of HIV-infected children, as well as for designing national policies on nutritional interventions in these children.

To explore anemia among children living with HIV and the role of iron therapy, we conducted a multi-centric study to examine the prevalence and risk factors of anemia and related micronutrient deficiencies such as iron, folic acid, vitamin B_12_ and vitamin A, among a cohort of children with perinatally-acquired HIV infection in southern India. We hypothesized that, in addition to nutritional factors including dietary intake, non-nutritional factors such as anemia of chronic inflammation play an important etiological role in childhood anemia in the context of HIV infection. We also examined the effect of anemia and therapeutic iron on growth, HIV disease progression and micronutrient deficiency status.

## Methods

### Study population

Children with perinatally acquired HIV infection aged between 2 and 12 years were screened and enrolled at three sites in South India, St John’s Hospital, Bangalore (a public-private partnership HIV center), National Institute for Research in Tuberculosis, Chennai (a public-funded research institute) and YRG Centre for AIDS Research and Education, Chennai (a private non-profit institution providing HIV care). Perinatally acquired HIV infection in children was indicated by history or documentation of one or both parents being HIV-infected. Both antiretroviral therapy (ART)-naïve (no perinatal or prior ART exposure) and ART-experienced children (on ART for at least 6 months) were included. Children younger than 2 years and older than 12 years were not included as they were likely to have varying nutrient requirements due to growth and pubertal changes. Children who had received any blood component transfusion within the past 6 weeks were excluded from the study.

### Ethical considerations

Written informed consent was obtained from the parent or legal guardian prior to enrolment. In addition, assent was obtained from children 8 years and older. The institutional review boards at all three participating sites approved the study.

### Study procedures

At the baseline visit, information on clinical history, socio-demographic details, current antiretroviral therapy, nutritional supplements and other medications was obtained. A complete physical examination was done, and HIV diagnosis documentation was verified. Anthropometric measurements included weight, height and mid-arm circumference. For younger children (aged between 2 and 4 years) recumbent length, instead of height was measured to the nearest 0.1 cm using a length of wooden board with a sliding foot piece. Height-for-age Z-scores, weight-for-age Z-scores and weight-for-height Z-scores were calculated (EpiInfo 3.3.2) based on the World Health Organization (WHO) Growth Standards of 2007 [12].

A 24-hour dietary recall was obtained from an interview with the caregiver and child, conducted by a research nutritionist. The quantity and the size of each food portion was estimated using standardized containers as described previously [13, 14], and subsequently analyzed using the Indian food composition tables to determine nutrient and caloric intake [15]. Dietary intake of children was compared with the recommended dietary allowance (RDA) and expressed as a percentage of RDA [16].

### Laboratory evaluation

The following routine laboratory tests were done: automated complete blood examination (Sysmex XT-2000i, Sysmex, Kobe, Japan), peripheral smear by manual examination and quantitative buffy coat assay for malarial parasites. CD4 T cell absolute counts and percentage values were measured using flow cytometry (FACSCalibur, Becton Dickenson Biosciences) and HIV viral load was performed using Real Time PCR (Abbot RealTime HIV-1, Abbott Park, IL, USA). Stool samples were processed into a direct saline and iodine wet mount and examined microscopically to detect intestinal parasites. Serum folate, vitamin B_12_, serum iron, transferrin and ferritin levels were measured by electrochemiluminescence using Roche Cobas 6000 (Roche Diagnostics Pvt. Ltd, Basel, Switzerland). Serum soluble transferrin receptor (sTfR), retinol binding protein levels and high-sensitivity C-reactive protein levels were assayed by immunonephelometry using BN ProSpec, Siemens Ltd (Siemens, Erlangen, Germany).

### Definitions

Anemia was categorized based on the WHO criteria for definition of anemia and was stratified based on age (children aged 6–59 months, hemoglobin (Hb) concentration <11.0 g/dl; 5–11 years, Hb <11.5 g/dl; ≥12 years, Hb <12.0 g/dl)[17]. Severe anemia was defined as Hb <7.0 g/dl for children aged 6–59 months; and Hb <8.0 for those 5 years and older [17]. Iron deficiency was defined as soluble transferrin receptor-log ferritin index (sTfR/lf) ≥1.5; [18]. Presence of inflammation was indicated by ultrasensitive C-Reactive Protein (CRP) >1.0 mg/dl. Anemia of inflammation was defined as sTfR/lf ≤ 1.5 plus CRP >1.0 mg/dl [18]. Vitamin B_12_ deficiency was defined as serum B_12_ ≤ 210 pg/mL [19] and folate deficiency as serum folate <140 ng/ml [19]. Vitamin A deficiency was indicated by retinol binding protein (RBP) as <0.7 μmoles/L [20], which is a known sensitive and specific marker for vitamin A deficiency in the context of HIV infection and malnutrition [21].

### Follow-up visits and iron therapy

Children were assessed every 3 months for one year. Clinical and anthropometric measurements were collected every 3 months, while laboratory assessments took place every 6 months. The study did not include any intervention, however clinical guidelines from the World Health Organization were followed by the clinician for iron therapy [22]. For treatment, a colloidal form of iron containing ferric hydroxide with elemental iron of 53 % (Tonoferon, East India Pharmaceutical Works Limited) prescribed at a dose of 3 mg/kg body weight was used. Iron was dispensed for 3 months, following which iron was continued for 6 months if the children remained anemic.

### Statistical analysis

Frequency distributions of anemia and micronutrient deficiencies were calculated using simple proportions and compared using chi square tests. Bivariate analysis of explanatory variables and anemia prevalence was followed by logistic regression and multivariate analysis of all identified covariates that were statistically significant at a level of *p* < 0.05 to describe the final model of predictors of anemia. Chi square tests were used to explore categorical variables, and odds ratios with 95 % confidence intervals expressed the association between the variables. All statistical analysis was performed using Stata v13.0 software. All tests were 2-tailed and were considered statistically significant at a level of *p* < 0.05.

## Results

### Baseline characteristics

Between February 2011 and August 2012, 286 children were screened at all participating sites. Parents’ HIV status was unknown in 7 children, there were 34 children who did not fulfill age criteria and were either below 2 years or older than 12 years, duration of ART was less than 6 months in 2 children during the period of recruiting, and caregivers of 3 children refused to give consent. The final number of children recruited for the study was 240 and were equally distributed among the three sites. Among the 240 children enrolled in the study, mean age was 7.7 years (SD 2.6), and there were 131 males (54.6 %). Distribution of WHO Clinical staging among the children was as follows: stage 1 and 2: 80.8 %; stage 3 and 4: 19.2 %. Median CD4 percentage was 25 % (IQR 18, 33), median absolute CD4 count was 773 cells/mm^3^ (IQR = 507, 1251) and proportion of children with baseline CD4 < 350 cells/mm^3^ was 27/240 (11.3 %). There was a high prevalence of malnutrition at baseline; proportion of children with stunting (height for age Z score HAZ < −2) was 40.0 %; those underweight (weight for age Z score WAZ < −2) was 45.4 %, and those with wasting (weight for height Z score WHZ < −2) was 23.3 %, and those children with low BMI (body mass index Z score BMIZ < −2) was 29.2 %. The proportion of children on ART at baseline was 104/240 (43.3 %). ART regimens included zidovudine or stavudine, with lamivudine and nevirapine, efavirenz or lopinavir/ritonavir. The prevalence of intestinal helminthic infestation was 28/240 (11.7 %) in this population, and included *Ascaris lumbricoides (11/28)*, *Giardia lamblia (7/28)*, *Enterobius vermicularis (7/28)*, *Trichomonas hominis (2/28) and Entamoeba histolytica (1/28).*

### Anemia prevalence and associations

Anemia was prevalent in 113/240 children (47.1 %), while severe anemia was seen in 16/240 (6.7 %). Overall iron deficiency was prevalent in 154/240 (64.4 %). Vitamin A deficiency prevalence was 43/240 (17.9 %), while folate and vitamin B_12_ deficiencies were 1/240 (0.4 %) and 15/240 (6.3 %) respectively. Risk factors for anemia in a bivariate model included stunting, CD4 < 25 %, detectable viral load ≥400 copies/ml and absence of ART and vitamin A deficiency (Table 1). When all the potential risk factors were added to a multivariate model, we found that significant independent risk factors for anemia were stunted status (OR 1.9, 95%CI 1.1-3.4), low CD4 count (OR 3.2, 95%CI 1.8-5.7), detectable viral load (OR 2.4, 95%CI 1.1-5.4) and vitamin A deficiency (OR 2.5, 95%CI 1.1-5.6) (Table 1). Drugs such as co-trimoxazole did not have an impact on anemia prevalence.

**Table 1:** Bivariate and multivariate analysis of risk factors of anemia in HIV

Parameters	Anemic children	Non-anemic children	Bivariate analysis		Multivariate analysis	
	n = 113	n = 128	OR (95 % CI)	*p*	OR (95 % CI)	*p*
Younger age (<6 years)	28 (24.8)	23 (18.1)	1.5 (0.8-2.8)	0.209	-	-
Underweight (Weight-for age Z score < − 2)	57 (50.4)	51 (42.2)	1.5 (0.9-2.5)	0.111	-	-
Stunted (Height-for age Z score < − 2)	53 (46.9)	42 (33.1)	1.8 (1.1-3.0)	0.029*	1.9 (1.1-3.4)	0.034*
Not on antiretroviral therapy	81 (71.7)	55 (43.3)	3.3 (1.9-5.7)	<0.001*	1.4 (0.7-3.0)	0.359
Low CD4% (<25 %)	75 (66.4)	41 (32.3)	4.1 (2.4-7.1)	<0.001*	3.2 (1.8-5.7)	<0.001*
Viral load ≥ 400 (copies/ml)	92 (82.4)	67 (52.8)	3.9 (2.2-7.1)	<0.001*	2.4 (1.1-5.4)	0.035*
Intestinal helminth infestation	15 (13.2 %)	13 (10.2)	1.1 (0.6-3.1)	0.601	-	-
Inflammation (ultrasensitive CRP > 1.0) mg/dl)	66 (58.4)	69 (54.8)	1.2 (0.7-2.0)	0.570	-	-
Iron deficiency (soluble transferrin receptor-log ferritin index (sTfR/lf) ≥1.5)	74 (65.5)	80 (62.9)	1.1 (0.7-1.9)	0.688	-	-
B_12_ deficiency (serum B_12_ ≤ 210 pg/mL)	11 (9.8)	4 (3.2)	3.3 (1.0-10.6)	0.047	-	-
Vit A deficiency (retinol binding protein (RBP) <0.7 μmoles/L)	30 (26.6)	13 (10.2)	3.2 (1.6-6.0)	0.001*	2.5 (1.1-5.6)	0.022*

* refers to statistical significance at a level or p > 0.05

### Etiology of anemia

**Fig. 1:**
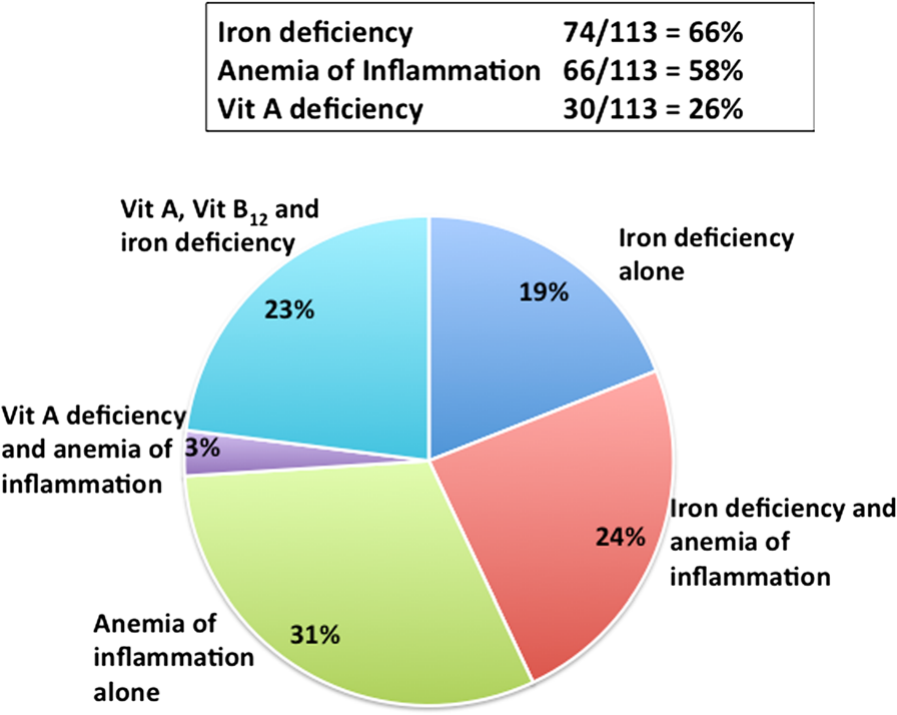
Etiology of anemia in HIV. Proportions of micronutrient deficiency and inflammation contributing towards the etiology of anemia among HIV-infected children

Among those children with anemia, iron deficiency was the commonest micronutrient deficiency; 74/113 (65.5 %) had iron deficiency anemia, Vitamin A deficiency was seen in 30/113 (26.6 %), while 1/112 (0.9 %) and 9/112 (8.0 %) had folate and vitamin B_12_ deficient respectively. Anemia of inflammation was seen in 66/113 (58.4 %) of anemic children. There were several overlapping micronutrient deficiencies as well as evidence of inflammation that was associated with anemia (Fig. 1). At the initial testing stage, there were no children whose anemia could be attributed to zidovudine. The presence of ART at baseline did not impact the etiology pattern of anemia.

### Dietary intake

Median intake of nutrients expressed as percentage of RDA was 36 % for iron, and 72 % for energy, indicating that on an average, most of the children were obtaining less than half of the recommended dietary allowance for iron. The proportion of children getting less than minimum RDA (at least 75 % RDA) for iron was as high as 79.9 % and for energy was 43.3 % (Table 2). These proportions did not change significantly over the 1-year follow-up period.

**Table 2:** Dietary intake among children with HIV

Nutrient	Median intake in our population (IQR)	Recommended Dietary Allowance, ICMR, 2010		Children obtaining <75 % of RDA n (%)
Energy (kcal/day)	1275 (966, 1615)	4–6 yr:	1350	104 (43.3)
	1362 (1011, 1695)	7–9 yr:	1690	
	1571 (1146, 1930)	Boys 10–12 yr:	2190	
	1487 (1024, 1720)	Girls 10–12 yr:	2110	
Protein (g/day)	37.6 (25.9, 49.7)	4–6 yr:	20.1	104 (43.3)
	40.0 (31.0, 53.6)	7–9 yr:	29.5	
	47.0 (37.6, 67.7)	Boys 10–12 yr:	39.9	
	41.0 (29.5, 53.6)	Girls 10–12 yr:	40.4	
Fat (g/day)	32.4 (22.8, 45.8)	4–6 yr:	25	49 (20.4)
	39.1 (26.4, 52.5)	7–9 yr:	30	
	45.4 (28.7, 56.0)	Boys 10–12 yr:	35	
	37.2 (28.7, 52.9)	Girls 10–12 yr:	35	
Iron (mg/day)	7.9 (5.1, 10.4)	4–6 yr:	13	187 (77.9)
	8.4 (5.8, 11.9)	7–9 yr:	16	
	9.6 (6.9, 13.0)	Boys 10–12 yr:	21	
	9.0 (5.9, 11.9)	Girls 10–12 yr:	27	
Vitamin A (retinol, mg/day)	748.6 (427.4, 1069.3)	1–17 yr:	600	128 (53.3)
B_12_ (mg/day)	1.4 (0.8, 2.3)	1–17 yr:	0.2–1.0	47 (19.6)
Folate (mg/day)	148.3 (108.8, 208.4)	4–6 yr:	100	35 (14.6)
		7–9 yr:	120	
		10–12 yr:	140	

The dietary intakes of macro- and micronutrients are presented in relation to standard recommended dietary allowances (RDA) for Indian children. Age and gender-stratified values are indicated. The last column refers to the proportion of children in the study who receive less than 75 % of standard RDA for each nutrient studied

*IQR* interquartile range, *ICMR* Indian Council of Medical Research

### Follow-up and iron therapy

Follow-up data were available for 194/240 (80.8 %) children who returned for their 6-month and 12 month visit. There were 18 children who were transferred out to a different ART center and could not return for follow-up visits, 8 children died, 1 child whose caregiver withdrew consent, and 19 were lost-to-follow-up. During the follow-up period, ART was initiated among 42 children, and 32 received zidovudine-based treatment. Among these, 3 children developed zidovudine-related bone marrow suppression with severe anemia and were switched to stavudine or abacavir-based ART.

Among 113 children who were initially anemic, 77 children received therapeutic iron after the baseline visit. Of the remaining 35 children who did not receive iron, 25 children had haemoglobin >11 g/dl and were not considered “anemic” and the clinical decision to give iron was not taken. Two children died within 1 month of baseline visit, and the remaining 8 children were amongst those who were lost-to-follow-up as described earlier. Assessment of adherence to iron, assessed by telephone or personal contact during the clinic visit, indicated that 70 % of the children reported 100 % adherence, 20 % missed 1–2 weeks of therapy, and 10 % missed over 2 weeks of iron therapy. Mild adverse effects to iron were reported by 17/77 (22 %), and included dark colored stools, nausea, diarrhea or constipation and mild abdominal discomfort. All these were minor side effects that diminished after 2–3 weeks of first reporting. The number of hospitalizations for intercurrent infections (pneumonia, tuberculosis and other infections) was similar in both groups (3 among iron supplementation group and 2 in the non-iron group). No malaria was reported among these children.

### Effect of iron therapy

Among children who received iron for 3 months, median hemoglobin increased from 10.4 g/dl to 10.9 mg/dl (Table 3). Hemoglobin change was maximum after 1 year, and increased to 11.3 mg/dl among children who received iron for up to 6 months. Children who were on ART plus iron had a greater hemoglobin increase compared to children who were on ART alone, without iron (Hb change 1.3 versus 0.4 gm/dl respectively, *p* = 0.009). The prevalence of iron deficiency also decreased from 68.1 to 49.2 % (*p* = 0.04) among those who received iron. In addition, this group also showed a decreased trend in clinical severity; severe WHO clinical stage (Stage 3, 4) decreased from 25.7 % at baseline to 10.9 % at 1 year of follow-up. A smaller decrease in clinical severity stage (16.7–12.5 %) was seen among those who did not receive iron. There was no significant change in the presence of chronic inflammation among those who received iron supplements. Iron did not independently affect growth or CD4 parameters; overall improvement of WAZ and HAZ were seen over one year irrespective of iron supplements.

**Table 3:** Change in parameters following iron therapy

Parameters	Timing (from enrolment)	Received iron supplements	Iron supplement not received	*p*
Hemoglobin (g/dl)	Baseline	10.4	12.0	0.003*
	3 months	10.9	12.0	0.04*
	1 year	11.3	11.9	0.2
Weight-for-age Z score	Baseline	−2.2	−1.9	0.08
	1 year	−1.9	−1.8	0.2
Height-for-age Z score	Baseline	−1.6	−1.5	0.2
	1 year	−1.3	−1.7	0.05
CD4%	Baseline	21.0	20.5	0.6
	1 year	26.5	26.0	0.7
Prevalence of advanced WHO clinical stage (Stage 3, 4) (%)	Baseline	25.7	16.7	0.04*
	1 year	10.9	12.5	1.0
Fe deficiency prevalence (STfR/lf) >1.5) (%)	Baseline	68.1	62.7	0.04*
	1 year	49.2	58.0	1.0
Chronic inflammation (CRP > 1) (%)	Baseline	62.9	52.4	0.05
	1 year	57.9	40.0	0.06

Children (*n* = 194) were followed for one year, and the change in parameters are compared between those who received and did not short-course iron supplementation

*WHO* World Health Organization, *sTfR/lf* soluble transferrin receptor-log ferritin index, *CRP* C-reactive protein

* refers to statistical significance at a level or p > 0.05

## Discussion

Our study revealed a significant prevalence of anemia among children with perinatally-acquired HIV, with major determinants being iron deficiency, chronic inflammation and vitamin A deficiency in this population. Anemia was associated with stunting and poor disease control (detectable viral load and low CD4 counts). The use of therapeutic iron appeared to be safe in these children, and resulted in significant improvement in the degree of anemia, iron deficiency and clinical progression in this population.

Other recent studies have indicated that anemia continues to be a problem in the post-ART era. In many low-income settings, unattended malnutrition, helminthic infections, poor dietary intake and food insecurity may often abrogate the positive effects of ART [23]. Prevalence of anemia and undernutrition in Nigeria in the setting of ART was reported as 70 %[9]. The IeDEA West Africa collaboration study involving children from seven countries indicated that although anemia was less common among those on ART, severe malnutrition is linked to severe anemia despite the presence of ART [24]. From the TREAT Asia Pediatric HIV Observational Database, among 1648 children on ART, incidence of new onset anemia was low in the setting of ART, but was associated with malnutrition, advanced disease state and the use of zidovudine-containing regimens [25].

Major nutritional determinants of anemia include micronutrient deficiencies such as iron and vitamin A, folate and B_12_.[26, 27]. Few studies in India have scrutinized the role of different micronutrients in the development of anemia, and our results showing the role played by iron deficiency, vitamin A deficiency along with chronic inflammation are useful in determining targeted nutritional strategies for management. Although our study did not indicate any significant association with intestinal parasitic infestation, the triple burdens of HIV, intestinal parasitic infections and anemia often coexist in children, and are often associated with lower CD4+ T cell levels in HIV infected children [28]. Further, stool examination for ova and cysts is notoriously insensitive and the prevalence of soil-transmitted helminth infestation was probably under-estimated. Other studies of Indian children have shown prevalence ranging from 13 to 68 % [29] with lower prevalence reported from urban areas.

The alliance between HIV infection and iron deficiency is incompletely characterized. Several studies indicated that children infected with HIV have iron deficiency less frequently than children without HIV infection [30–32], and a review on iron status in children with HIV indicated that the prevalence of iron deficiency is low in both high and low-income areas [33]. However, the chronic inflammatory state frequently present during HIV infection may lead to underestimation of iron deficiency in this population, because markers of iron status such as ferritin are elevated in chronic inflammation [34, 35]. On the other hand, inflammation-induced hypoferraemia may result in functional iron deficiency, which renders iron unavailable for erythropoiesis. The resulting anemia also makes iron unavailable for infective organisms that require iron for growth and proliferation, and results in anemia of inflammation [18].

Persistent anemia in the setting of ART can be a reflection of underlying pro-inflammatory pathways induced by HIV [36]. A study in a large cohort of adults and children in Uganda and Zimbabwe showed that while ART significantly reduced the prevalence of anemia two years after initiating therapy, 13 % of the population continued to be anemic, attributable to the chronic inflammatory state [37]. The role of chronic inflammation in causing anemia has been highlighted in several studies of HIV-infected individuals. Among 299 Thai and Cambodian children with HIV, although anemia was prevalent among 50 % of the children, iron deficiency prevalence, as measured by ferritin levels was less than 3 % overall; and most of the anemia was attributed to anemia of inflammation and thalassemia trait [38]. Since sTfR is a biomarker that is less influenced by inflammatory parameters, we leveraged this property to obtain a more accurate appraisal of iron deficiency in our population. This may explain the high prevalence of iron deficiency identified in this setting. There is little doubt that nutritional anemia, including iron deficiency anemia is a major co-morbidity in children with HIV. Iron deficiency is also strongly associated with impaired neurobehavioral development [39] and needs to be addressed in this population. In malaria-endemic places, this equipoise is often disrupted, and iron deficiency appears to be protective against clinical malaria in children [40], with iron supplementation being associated with an increased risk of malaria and hospitalization or death [11].

Our results indicate that therapeutic iron taken for a period of 3–6 months appear safe with only minor transient adverse effects, and with no increase in incidence of malaria or hospitalization. These results may not be generalizable to a malaria-endemic setting where the risks of iron supplementation have been consistently noted. The use of therapeutic iron in our study population also showed a beneficial effect on growth and disease progression. In a placebo-controlled randomized controlled trial in Malawi, researchers showed that iron supplementation in anemic HIV-infected children had beneficial effects on hemoglobin, anemia prevalence and CD4 counts at 6 months but increased the risk of malaria [41]. This trial also noted that among iron-deficient children, iron theapy was associated with a reduced risk of progression to AIDS. Importantly, our study indicated that a combination of iron and ART worked well to improve hemoglobin, rather than ART alone [42].

Dietary sources of iron among these south Indian children were inadequate. An appalling 80 % of the children in this study received less iron than the minimum RDA for age (defined as <75 % RDA). A prospective study from South Africa indicated insufficient dietary iron among children; although anemia improved over the 18-month time period with supplementation, iron deficiency paradoxically increased from 15 % at baseline to 37 % during the same period, suggesting that low dose iron supplementation along with dietary increase would be a useful strategy [43]. In low-income settings, low dietary iron intake and poor bioavailability of dietary iron may be a challenge. Some inexpensive strategies to counter this can include use of unrefined sugar and local unpolished cereals, measures to improve the bioavailability of iron such as soaking cereals and legumes, and combining cereals and legumes with food sources of vitamin C, such as fresh fruit and vegetables [44].

Our study may be limited by the relatively small sample size, and the lack of a randomized controlled intervention to assess the effect of iron therapy. In addition, the use of ferritin as a marker can result in non-representative values for iron deficiency in the setting of chronic inflammation; however we have used, along with ferritin, sTfR for defining iron deficiency, which reflects the degree of tissue iron supply as well as iron stores [45]. We did not assess the presence of hemoglobinopathies in this setting, and hence this data is unknown. Previous studies have found the prevalence of alpha and beta-thalassemia to be low in this geographic area [46]; hence the non-inclusion of this parameter in our assessment was unlikely to have influenced anemia prevalence or etiology.

## Conclusions

Our study highlights the finding that Indian children with HIV have significant presence of anemia that is orchestrated by iron deficiency, although vitamin A deficiency and chronic inflammation also play a role. Dietary sources of iron remain highly inadequate. The use of standard WHO-recommended therapeutic iron particularly for durations of up to 6 months appears to be safe in this setting, and is associated with beneficial effects on anemia, iron deficiency and HIV disease progression. Further clinical and cost-effectiveness studies of iron therapy will be useful in streamlining policy decisions on anemia management among HIV-infected children in non-malaria endemic regions.

## Abbreviations

HIV: Human Immunodeficiency Virus
ART: Anti-Retroviral Therapy
WHO: World Health Organization
RDA: Recommended Dietary Allowance
Hb: Hemoglobin
sTfR/lf: soluble transferrin receptor-log ferritin index
CRP: C-Reactive Protein
RBP: Retinol Binding Protein
OR: Odds Ratio
WAZ: Weight-for-Age Z Score
HAZ: Height-for-Age Z Score
IQR: Interquartile range
ICMR: Indian Council of Medical Research
